# Establishment and Verification of a Bagged-Trees-Based Model for Prediction of Sentinel Lymph Node Metastasis for Early Breast Cancer Patients

**DOI:** 10.3389/fonc.2019.00282

**Published:** 2019-04-16

**Authors:** Chao Liu, Zeyin Zhao, Xi Gu, Lisha Sun, Guanglei Chen, Hao Zhang, Yanlin Jiang, Yixiao Zhang, Xiaoyu Cui, Caigang Liu

**Affiliations:** ^1^Department of Breast Surgery, Shengjing Hospital of China Medical University, Shenyang, China; ^2^Sino-Dutch Biomedical and Information Engineering School, Northeastern University, Shenyang, China; ^3^Department of Urology Surgery, Shengjing Hospital of China Medical University, Shenyang, China

**Keywords:** breast cancer, sentinel lymph nodes, metastasis prediction, model, bagged-trees

## Abstract

**Purpose:** Lymph node metastasis is a multifactorial event. Several scholars have developed nomograph models to predict the sentinel lymph nodes (SLN) metastasis before operation. According to the clinical and pathological characteristics of breast cancer patients, we use the new method to establish a more comprehensive model and add some new factors which have never been analyzed in the world and explored the prospect of its clinical application.

**Materials and methods:** The clinicopathological data of 633 patients with breast cancer who underwent SLN examination from January 2011 to December 2014 were retrospectively analyzed. Because of the imbalance in data, we used smote algorithm to oversample the data to increase the balanced amount of data. Our study for the first time included the shape of the tumor and breast gland content. The location of the tumor was analyzed by the vector combining quadrant method, at the same time we use the method of simply using quadrant or vector for comparing. We also compared the predictive ability of building models through logistic regression and Bagged-Tree algorithm. The Bagged-Tree algorithm was used to categorize samples. The SMOTE-Bagged Tree algorithm and 5-fold cross-validation was used to established the prediction model. The clinical application value of the model in early breast cancer patients was evaluated by confusion matrix and the area under receiver operating characteristic (ROC) curve (AUC).

**Results:** Our predictive model included 12 variables as follows: age, body mass index (BMI), quadrant, clock direction, the distance of tumor from the nipple, morphology of tumor molybdenum target, glandular content, tumor size, ER, PR, HER2, and Ki-67.Finally, our model obtained the AUC value of 0.801 and the accuracy of 70.3%.We used logistic regression to established the model, in the modeling and validation groups, the area under the curve (AUC) were 0.660 and 0.580.We used the vector combining quadrant method to analyze the original location of the tumor, which is more precise than simply using vector or quadrant (AUC 0.801 vs. 0.791 vs. 0.701, Accuracy 70.3 vs. 70.3 vs. 63.6%).

**Conclusions:** Our model is more reliable and stable to assist doctors predict the SLN metastasis in breast cancer patients before operation.

## Introduction

The incidence of breast cancer is the first in female malignant tumors, in which the highest incidence of breast cancer has been reported in Europe and the United States, however, in recent years, the incidence of breast cancer in China has annually increased (1, 2). Based on surgery as an important step in the treatment of breast cancer, in recent years, different individuals have never stopped exploration of a novel and optimum approach. Besides, NSABP-04, ASCOG-Z0011, and other tests have shown that for breast cancer patients with T1-T2 stage and clinical negative lymph node (cN0) during breast preservation surgery and total breast radiotherapy, axillary lymph node dissection (ALND) does not contain great benefits to the long-term survival of patients. As a result, sentinel lymph node biopsy (SLNB) has been gradually replaced with conventional ALND as a routine surgical method for early breast cancer patients (3–7). However, SLNB, as an invasive operation, leads to some postoperative complications. Although the corresponding incidence rate is lower than ALND, however, those complications should not be ignored. Moreover, SLNB has a high degree of professional requirement for physicians, and the richness of physician's experience directly affects the evaluation of the pathological status of sentinel lymph nodes (SLN).

In recent years, the concepts of precision medicine and individualized therapy have rapidly developed. We often ask the following questions: “Can SLNB be omitted for patients with lower probability of sentinel lymph node metastasis?” “OR For patients with higher probability of sentinel lymph node metastasis, can the SLNB to be skipped and the armpit treatment to be directly conducted?” OR “For patients with neoadjuvant chemotherapy, should we have an SLNB before neoadjuvant chemotherapy, or after it?” The state of axillary lymph nodes is not only a key factor in determining the mode of surgery, but also an important prognostic factor, and before surgery, patients often would like to know whether there is a transfer of SLN. With the idea of micro-non-invasive operation, several scholars have developed and used mathematical models to predict the pathological state of SLN before operation, in which the most important predictive model was designed in 2007 at Memorial Sloan-Caitlin Cancer Center (MSKCC; NY, USA). It has been shown that with a receiver operating characteristic (ROC) curve of 0.75, a proper level of prediction and discrimination can be achieved (8–24). However, there are differences in the sources of patients (ethnic, regional, cultural, economic conditions, disease awareness, etc.), surgical methods, pathological evaluation methods, and other factors. Hence, it is difficult to have a predictive model that can be universally used. The clinical and pathological parameters for the application of different predictive models are not the same. Hence, the purpose of our study was to analyze the clinical and pathological data of early breast cancer patients in a more comprehensive way, and establish a predictive model for sentinel lymph node pathology. Technically, nomogram which is now used worldwide use multiple logistic regression (MLR) to predict a binary outcome based on a combination of risk factors. This well-established method has a limitation in that it incorporates only a few independent variables so that the model can accurately predict risk in independent datasets, by avoiding over-fitting to the given datasets. Such prediction models should also tolerate missing values, which are common in clinical datasets (15). Thus, we use SMOTE-Bagged Tree as a core algorithm to cope with a greater number of variables and that provide accurate prediction and robustness against missing values. In addition to the variables analyzed by other scholars, we added some specific variables, such as breast glandular content, molybdenum target tumor morphology, and primary location of the tumor (clock direction and distance from the nipple). Our study for the first time presents a model to analyze these factors as well.

## Materials and Methods

### Patients

In this study, 633 patients with a clear state through sentinel lymph node examination (including lymph node biopsy and surgical treatment with ALND) were included. Analysis of clinical data involves the following variables: age, body mass index (BMI), tumor size, tumor location (quadrant, clock direction, distance from the nipple), clinical staging, pathological type, pathological classification, immunohistochemistry (IHC) [Estrogen receptor (ER), Progesterone receptor (PR), human epidermal growth factor receptor 2 (HER2), (Ki-67)], grading of tumor tissue, menopausal state, molybdenum target glandular content, and morphology and sentinel lymph node metastasis of molybdenum. The patient's information was derived from the Shengjing Hospital of China Medical University (Shenyang, China) during January 2011 to December 2014. The patients with early-stage breast cancer who met the following criteria were selected for treatment with SLNB: (1) diagnosis of breast cancer; (2) without receiving neoadjuvant chemotherapy; (3) the result for preoperative axillary lymph node to be negative, according to clinical and imaging examinations; (4) Primary diameter of tumor ranges at 0–5 cm; (5) Complete clinicopathological information; and (6) without pregnancy. Patients with incomplete data, metastasis of 3 and above axillary lymph nodes, distant metastasis, preoperative neoadjuvant chemotherapy, and radiotherapy were excluded. All patients involved in the present study signed a written informed consent form prior to their inclusion in the study. The study was approved by the Ethics Committee of Shengjing Hospital of China Medical University.

### Surgery Procedure

A standard breast cancer surgery was conducted on the basis of guidelines for the treatment of breast cancer patients in China. Surgery included primary tumor resection and lymph node biopsy (or ALND). The number and pathological status of SLN were detected after operation as well.

### Pathologic Evaluation

The Chinese breast cancer guidelines were used to evaluate surgical specimens. Tumors with > 10% nuclear-stained cells were considered positive for ER and PR. Ki67 expression > 20% was also considered positive. The HER-2 positivity was defined as a score of 3+ on IHC or amplification on FISH. If a pathologist scored the IHC 2+, the status of HER-2 was further investigated by FISH. In addition, the grade of breast cancer was determined by the Nottingham Histologic Scoring system. Tumor staging refers to the TNM staging method jointly conducted and published in form of the 8th edition by the International Anticancer Alliance (UICC) and the American Oncology Federation (AJCC) in 2018. The SLNs were step sectioned, stained with hematoxylin and eosin (H&E), and diagnosed by trained pathologists. Lymph nodes obtained after ALND were evaluated using a single H&E stained section from each node. Metastases were defined as the presence of a tumor deposit >0.2 mm in diameter in at least one lymph node.

### Location of the Tumor

**Figure 1:**
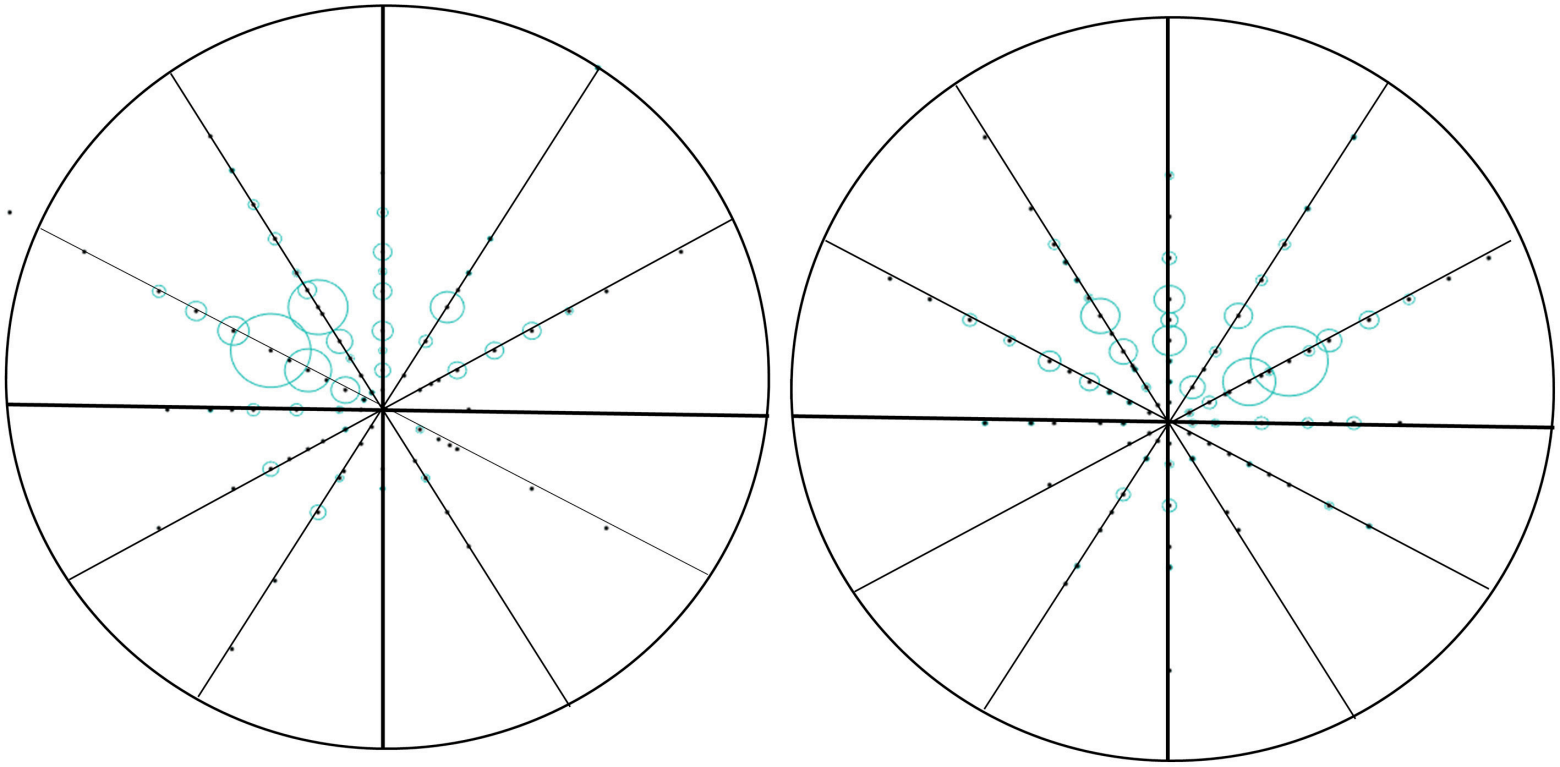
Vector diagram of the location of the tumor in breast. We counted the location of the tumor in the direction of the clock and the distance from the nipple (the factor of the clock in the left and right sides of the breast has been taken into account). The number of cases transferred at the same location is proportional to the radius, and then the nipple is the center of the circle, in which the distance is the radius of the mapping.

We use the polar coordinates to paint. We counted the location of the tumor in the direction of the clock and the distance from the nipple (the factor of the clock in the left and right sides of the breast has been taken into account). The number of cases transferred at the same location is proportional to the radius, and then the nipple is the center of the circle, in which the distance is the radius of the mapping (Figure 1).

### Statistical Analysis

In this study, MATLAB2018a was used for data processing, and statistical analysis was undertaken by using SPSS 25.0 statistical software (SPSS Inc., Chicago, IL, USA) and R software. The statistical significance level of the report was double-sided, and it was set to 0.05.

### Smote Algorithm Generates Data

There are a number of methods available to oversample a dataset used in a typical classification problem (using a classification algorithm to classify a set of images, given a labeled training set of images). The most common technique is known as SMOTE: Synthetic Minority Over-sampling Technique (25).

According to the natural occurrence of disease, breast cancer grows more in the outer upper quadrant. However, in the process of regular grouping, such case characteristic data is unbalanced (the proportion of the outer upper quadrant tumor is larger), which will affect the broad applicability of the model, so it is not appropriate to use the classifier to distinguish directly. Therefore, this study used the most appropriate smote algorithm to upsample the data to increase the balanced amount of data, reconstruct the training set, and obtain a relatively balanced training set.

The basic idea of smote algorithm is to analyze a few kinds of samples and add them to the dataset according to a few samples of synthetic samples, and the algorithm flow is as follows. (1) For each sample x in a few classes, the Euclidean distance is used as the criterion for calculating the distance of all samples in a few sample sets, and its k neighbor is obtained. (2) Set a sampling scale according to the unbalanced proportion of the sample to determine the sampling magnification n, for each minority sample X, from its K near neighbor randomly selected a number of samples, assuming that the selected neighbor is xn. (3) For each randomly selected near neighbor Xn, a new sample is constructed with the original sample according to the following formula.

$$\begin{array}{l} {\text{x}}_{new}=x+rand\left(0,1\right)\times \left(\tilde{\text{x}}-\text{x}\right) \end{array}$$

### Confusion Matrix

Confusion matrix is an important tool to evaluate the performance of classification model. A variety of evaluation indexes, such as true positive rate, false positive rate, true negative rate, false negative rate and accuracy, can be calculated by the obfuscation matrix. In particular, the confusion matrix distinguishes between false positives and false negatives of two different properties of miscalculation, which can be used to estimate the expected loss caused by miscalculation of the classification model. When the classification model returns the probability or score of each record belonging to the positive category, a obfuscation matrix can be obtained by specifying the threshold and making a positive judgment on all the probabilities or records that are rated above the threshold. By continuously changing the threshold value, different obfuscation matrices can be obtained, so that the ROC curve, and the performance of the classification model is evaluated and compared more comprehensively.

### Establishment of Predictive Models

First, we use the logistic regression method which was commonly used in other research centers to build prediction models, and then use the Bagged Tree algorithm to build prediction models. In the end we compared the results obtained by the two methods. The amplified data were classified using the Bagged Tree algorithm. It was used to analyze the following clinical candidate predictors: age, BMI, quadrant, clock direction, the distance of tumor from the nipple, morphology of tumor molybdenum target, glandular content, tumor size, ER, PR, HER2, and Ki-67. The trees in Bagged Trees were built on their own sampled datasets, and the training process was independent. The Bagged Tree algorithm extracts multiple random datasets to fit multiple decision tree models in order to improve model's performance. Each decision tree differs because of the subset data, and the final prediction results are determined based on the prediction of all trees (26). Accordingly, the versatility of predictions increases. The ROC curve was plotted, and the area under the curve (AUC) was here used to estimate the prediction accuracy of the model.

## Results

### Patient Characteristics

Our study included 633 patients who underwent sentinel lymph node examination, in which 35.8% of whom had sentinel lymph node metastasis. The descriptive characteristics of the model population are presented in Table 1. In fact, the characteristic data of lymph node metastasis in breast cancer were not balanced, and utilizing a classifier to directly distinguish was not appropriate. Therefore, the SMOTE was used to sample the breast cancer dataset and reduce the imbalance of the training set. An oversampling algorithm was also used to add new information to the unbalanced data. In our study, 633 cases of raw data were sampled by SMOTE, in which 169 new data were generated according to the characteristics of the original data. We analyzed the newly generated data and raw data (a total of 802 cases) together.

**Table 1:** Comparison of descriptive characteristics of the SLN+ group and SLN- group for the model.

**Variable**	**N**	**SLN+**		**SLN-**		***P*-value**
		**Population**	**%**	**Population**	**%**	
No. of cases	633	227	35.86	406	64.14	
Age						0.536
<40	52	17	32.69	35	67.31	
40–70	547	195	35.65	352	64.35	
>70	34	15	44.12	19	55.88	
AJCC T stage						0.149
T1a	1	0	0.00	1	100.00	
T1b	7	0	0.00	7	100.00	
T1c	349	118	33.81	231	66.19	
T2 < 3.0 cm	262	103	39.31	159	60.69	
T2 > 3.0 cm	14	6	42.86	8	57.14	
Left/Right breast						0.511
left	318	127	39.94	191	60.06	
right	315	100	31.75	215	68.25	
Neuroinvasion						0.102
Present	4	3	75.00	1	25.00	
Absent	629	224	35.61	405	64.39	
Lymphovascular invasion						0.232
Present	16	8	50.00	8	50.00	
Absent	617	219	35.49	398	64.51	
Tumor location						0.441
UOQ	313	122	38.98	191	61.02	
LOQ	47	15	31.91	32	68.09	
UIQ	127	38	29.92	89	70.08	
LIQ	42	16	38.10	26	61.90	
Central	104	36	34.62	68	65.38	
Tumor type						0.000
Ductal	609	211	34.65	398	65.35	
Lobular	10	4	40.00	6	60.00	
Mixed	10	10	100.00	0	0.00	
Mucinous	3	2	66.67	1	33.33	
Medullary	1	0	0.00	1	100.00	
Clinical stage						0.000
I	191	3	1.57	188	98.43	
II	440	223	50.68	217	49.32	
III	2	1	50.00	1	50.00	
Pathological grade						0.063
1	17	4	23.53	13	76.47	
2	582	205	35.22	377	64.78	
3	34	18	52.94	16	47.06	
ER						0.099
Present	481	181	37.63	300	62.37	
Absent	152	46	30.26	106	69.74	
PR						0.164
Present	427	161	37.70	266	62.30	
Absent	206	66	32.04	140	67.96	
HER2						0.534
Present	88	31	35.23	57	64.77	
Absent	545	196	35.96	349	64.04	
Ki67						0.960
Present	392	148	37.76	244	62.24	
Absent	241	79	32.78	162	67.22	
Breast gland content						0.214
<25%	99	31	31.31	68	68.69	
26–50%	184	42	22.83	142	77.17	
51–75%	305	113	37.05	192	62.95	
>75%	45	11	24.44	34	75.56	
Tumor morphology						0.366
Lump	378	130	34.39	248	65.61	
Mass with calcification	96	41	42.71	55	57.29	
No mass	159	56	35.22	103	64.78	
BMI						0.726
Too light	21	7	33.33	14	66.67	
Normal	313	108	34.50	205	65.50	
Overweight	299	112	37.46	187	62.54	

### Predictors of SLNM

**Figure 2:**
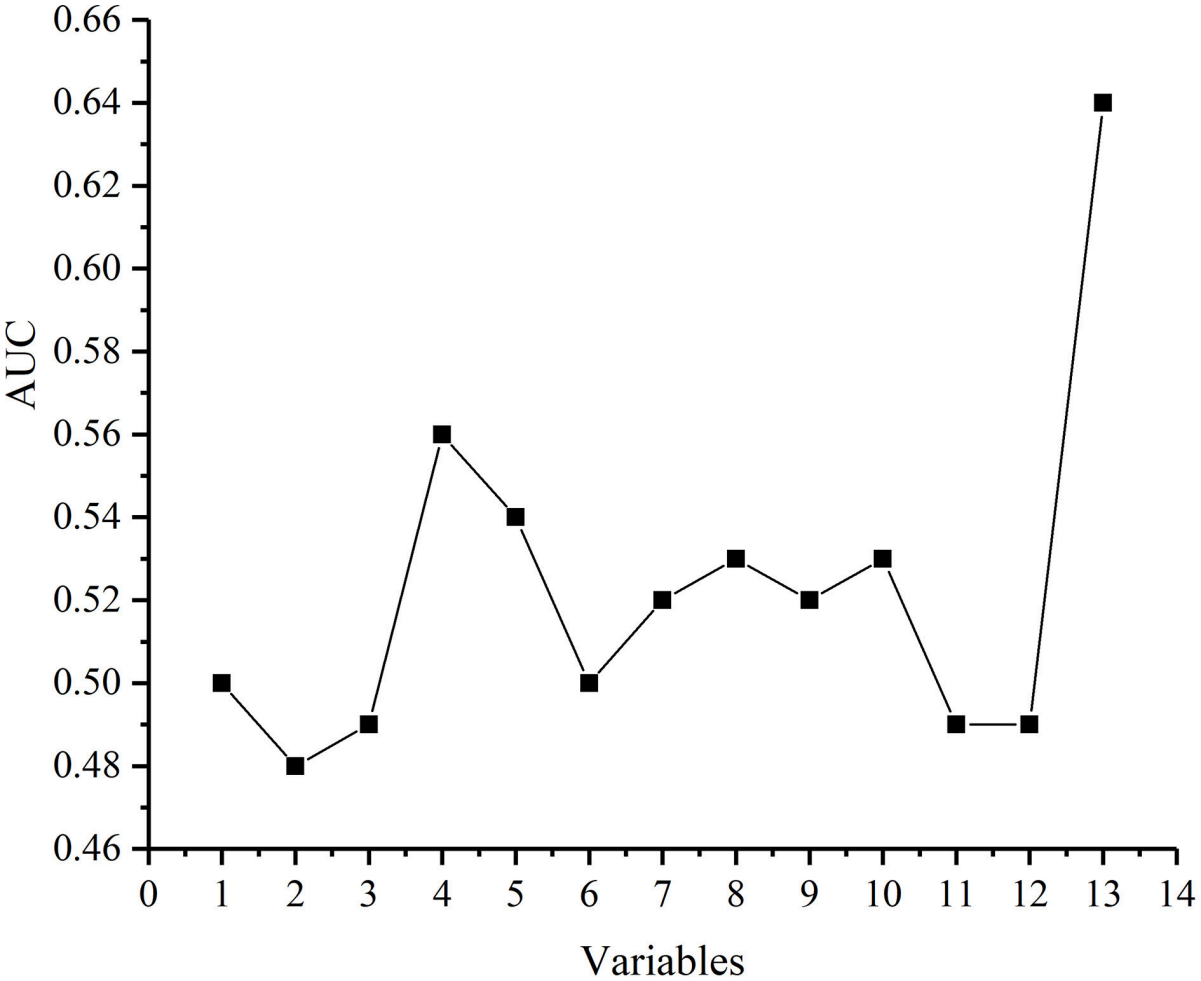
The ability of each variable to predict breast cancer SLN metastasis. Each point represents in turn: Age, body mass index(BMI), Quadrant, Clock direction, The distance of tumor from the nipple, Morphology of tumor molybdenum target, Glandular content, Tumor size, ER, PR, HER2,Ki-67, Vector (Clock direction and The distance of tumor from the nipple).

In the multi-factor analysis, we included the following variables associated with breast cancer SLN metastasis: age, BMI, quadrant, clock direction, the distance of tumor from the nipple, morphology of tumor molybdenum target, glandular content, tumor size, ER, PR, HER2, and Ki-67 (Figure 2).

### Construction and Validation of the Model by Logistic Regression

**Figure 3:**
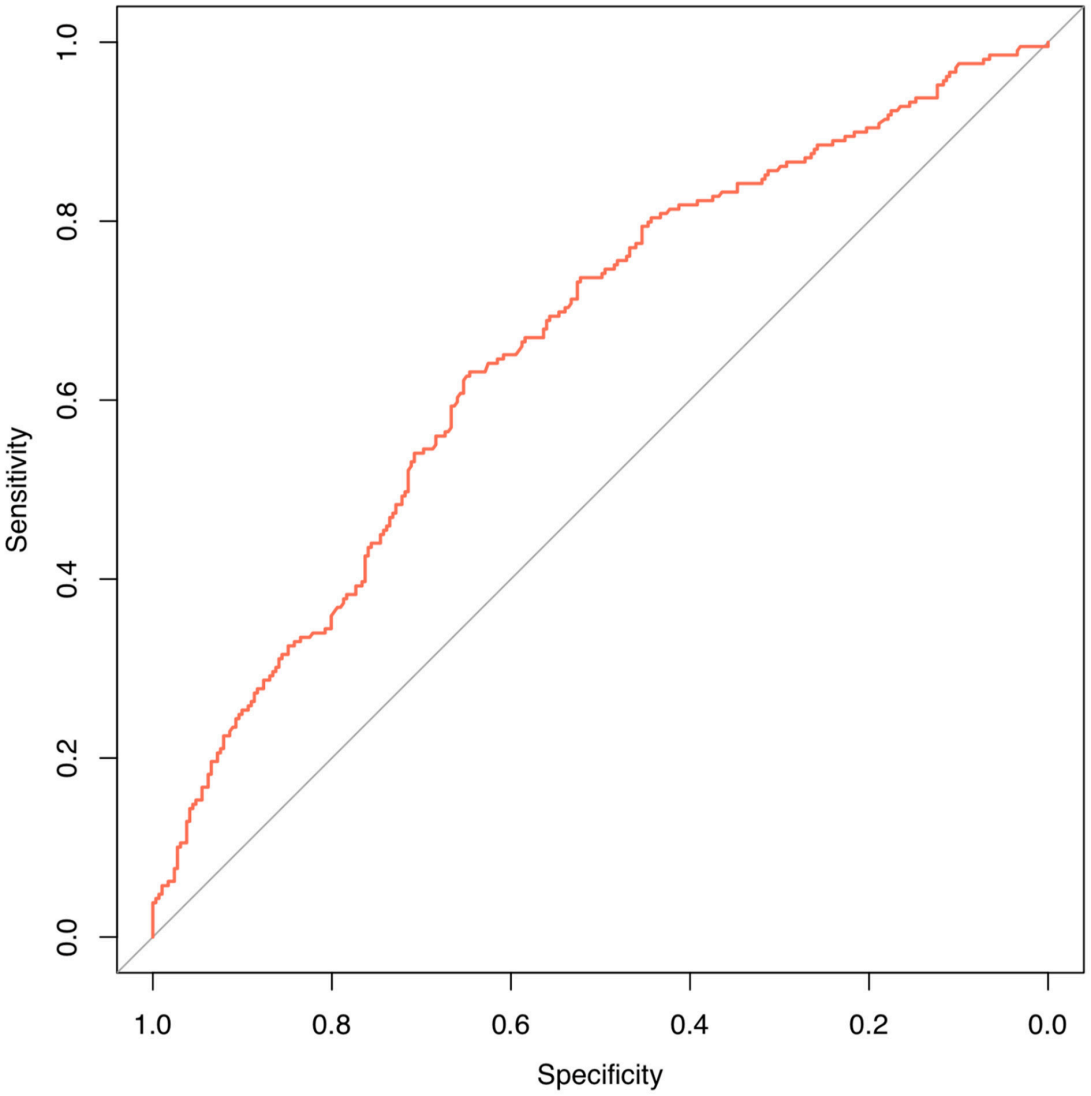
Internal validation using a ROC curve. Established model by logistic regression. The AUC value is 0.660.

**Figure 4:**
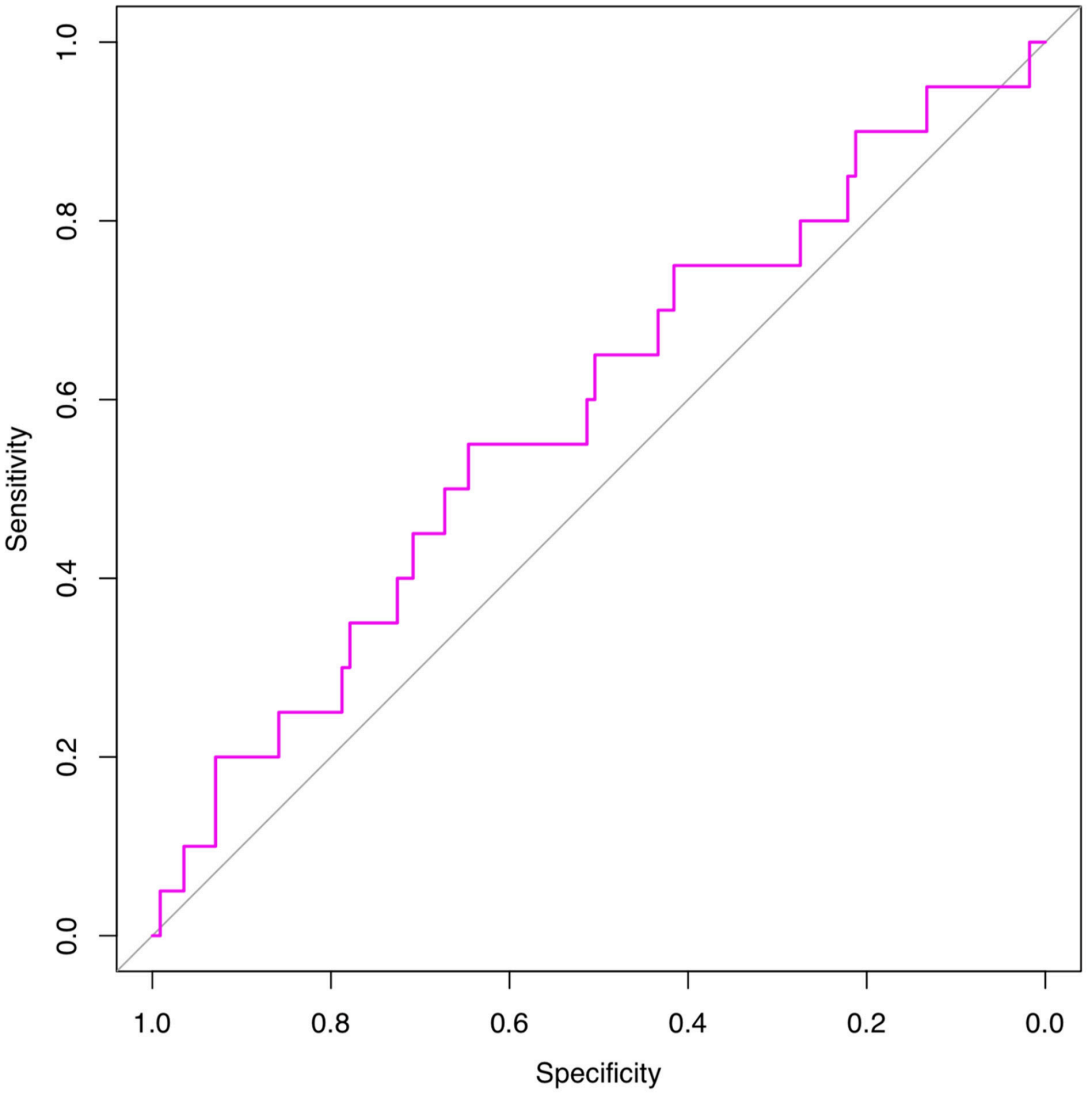
External validation using a ROC curve. Established model by logistic regression. The AUC value is 0.580.

We are grateful for your advice. Our study included 633 patients randomized into a modeling set (*n* = 500) and a validation set (*n* = 133). The clinicopathological characteristics of the patients did not differ significantly between the two groups (*P* > 0.05) in our study population (Table 2). The internal ROC curves in the modeling set and external ROC in the validation set were used to evaluate the model. In the modeling and validation groups, the AUC were 0.660 and 0.580 (Figures 3, 4).

**Table 2:** Comparison between modeling group and validation group by clinicopathological characteristics.

**Variable**	***N***	**Modeling**		**Validation**		***P*-value**
		**population**	**%**	**population**	**%**	
No. of cases	633	500		133		
Age						0.892
<40	52	42	80.80	10	18.20	
40–70	547	432	79.00	115	21.00	
>70	34	26	76.50	8	5.40	
Tumor location						0.178
UOQ	313	241	77.00	72	23.00	
LOQ	47	42	89.40	5	10.60	
UIQ	127	96	75.60	31	24.40	
LIQ	42	36	85.70	6	4.30	
Central	104	85	81.70	19	18.30	
Tumor type						0.439
Ductal	609	483	79.3	126	20.70	
Lobular	10	6	60.00	4	40.00	
Mixed	10	7	70.00	3	30.00	
Mucinous	3	3	100.00	0	0.00	
Medullary	1	1	100.00	0	0.00	
Clinical stage						0.270
I	191	157	82.20	34	17.80	
II	440	342	77.70	98	22.30	
III	2	1	50.00	1	50.00	
Pathological grade						0.899
1	17	13	76.50	4	23.50	
2	582	461	79.20	161	20.80	
3	34	26	76.50	8	23.50	
ER						0.353
Present	481	384	79.80	97	20.20	
Absent	152	116	76.30	36	23.70	
PR						0.234
Present	427	343	80.30	84	19.70	
Absent	206	157	76.20	49	23.80	
HER2						0.802
Present	88	71	80.70	17	19.30	
Absent	545	427	78.80	115	21.20	
Ki67						0.960
Present	392	148	37.76	244	62.24	
Absent	241	79	32.78	162	67.22	
Breast gland content						0.769
<25%	99	82	82.80	17	17.20	
26–50%	184	144	78.30	40	21.70	
51–75%	305	238	78.00	67	22.00	
>75%	45	36	80.00	9	20.00	
Tumor morphology						0.056
Lump	378	311	82.33	67	17.70	
Mass with calcification	96	70	72.90	26	27.10	
No mass	159	119	74.80	40	25.20	
SLN						0.073
Present	227	170	74.90	57	24.10	
Absent	406	330	81.30	76	18.70	

### Construction and Validation of the Model by SMOTE-Bagged Tree Algorithm

**Figure 5:**
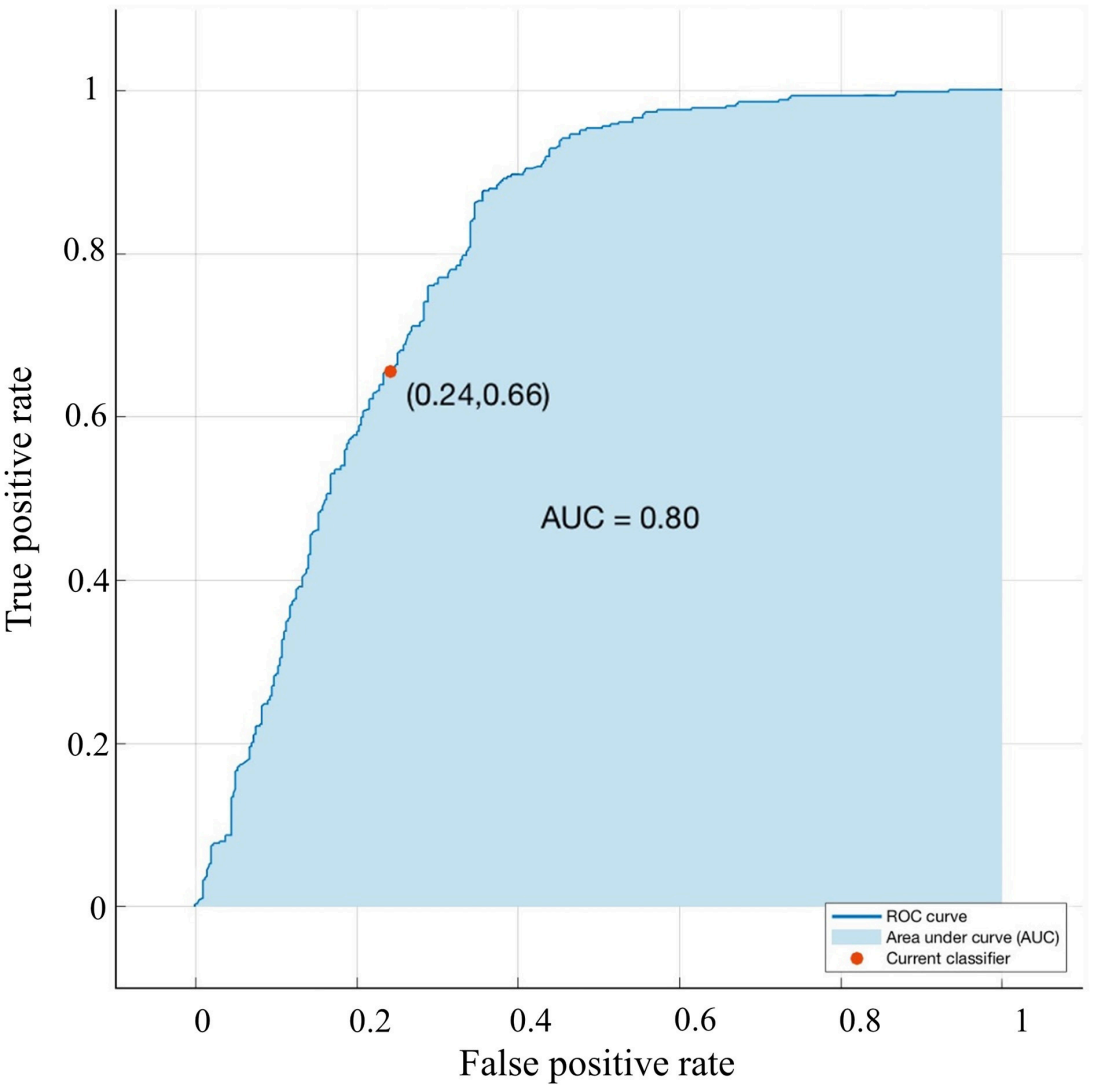
Validation using a ROC curve. Established model by Smote-Bagged-tree and used Vector combining quadrant method to analyze. The location of the tumor. The AUC value is 0. 801.

**Figure 6:**
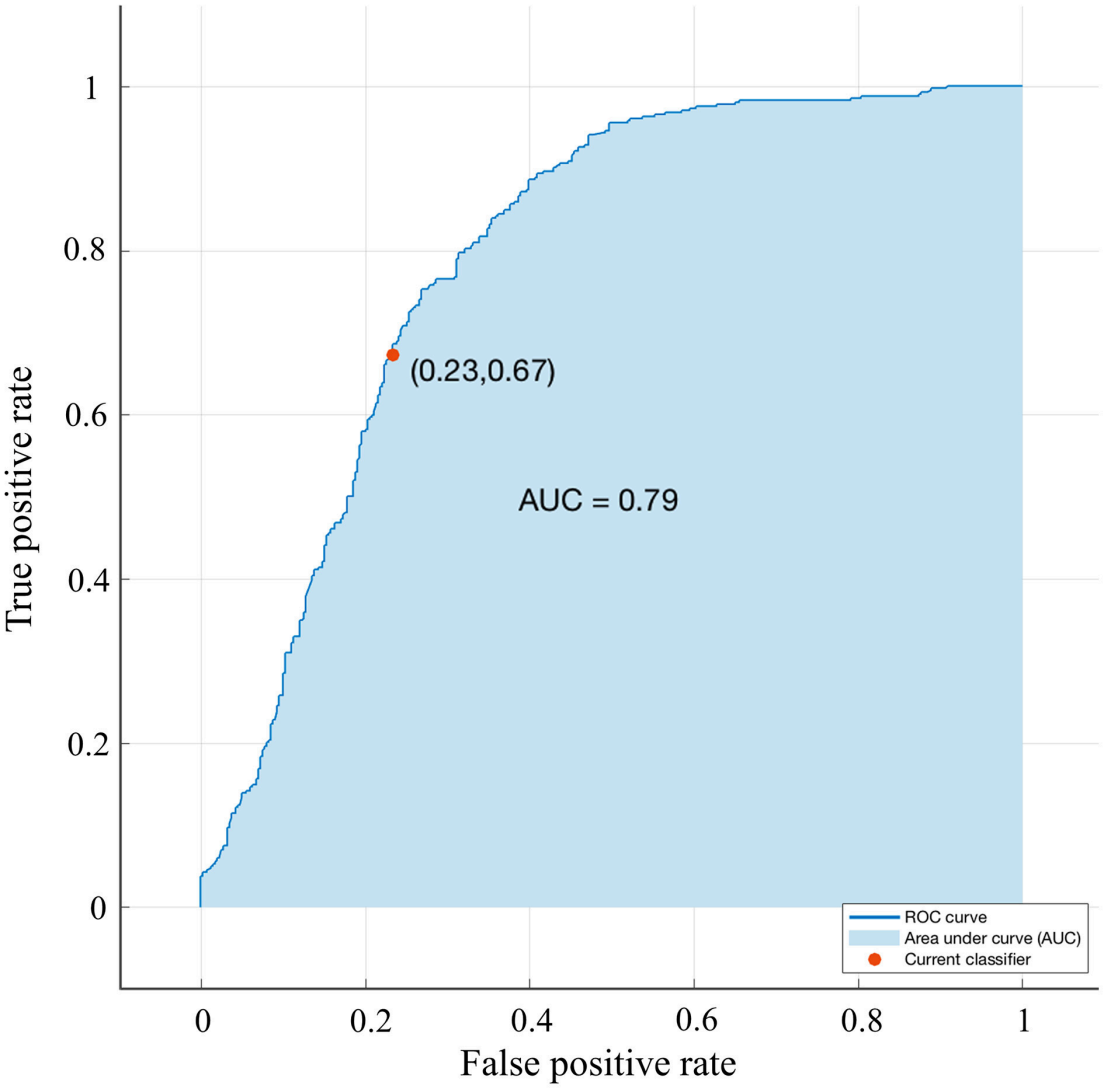
Validation using a ROC curve. Established by Smote-Bagged-tree and used Simply using vector method to analyze the location of the tumor. The AUC value is 0.791.

**Figure 7:**
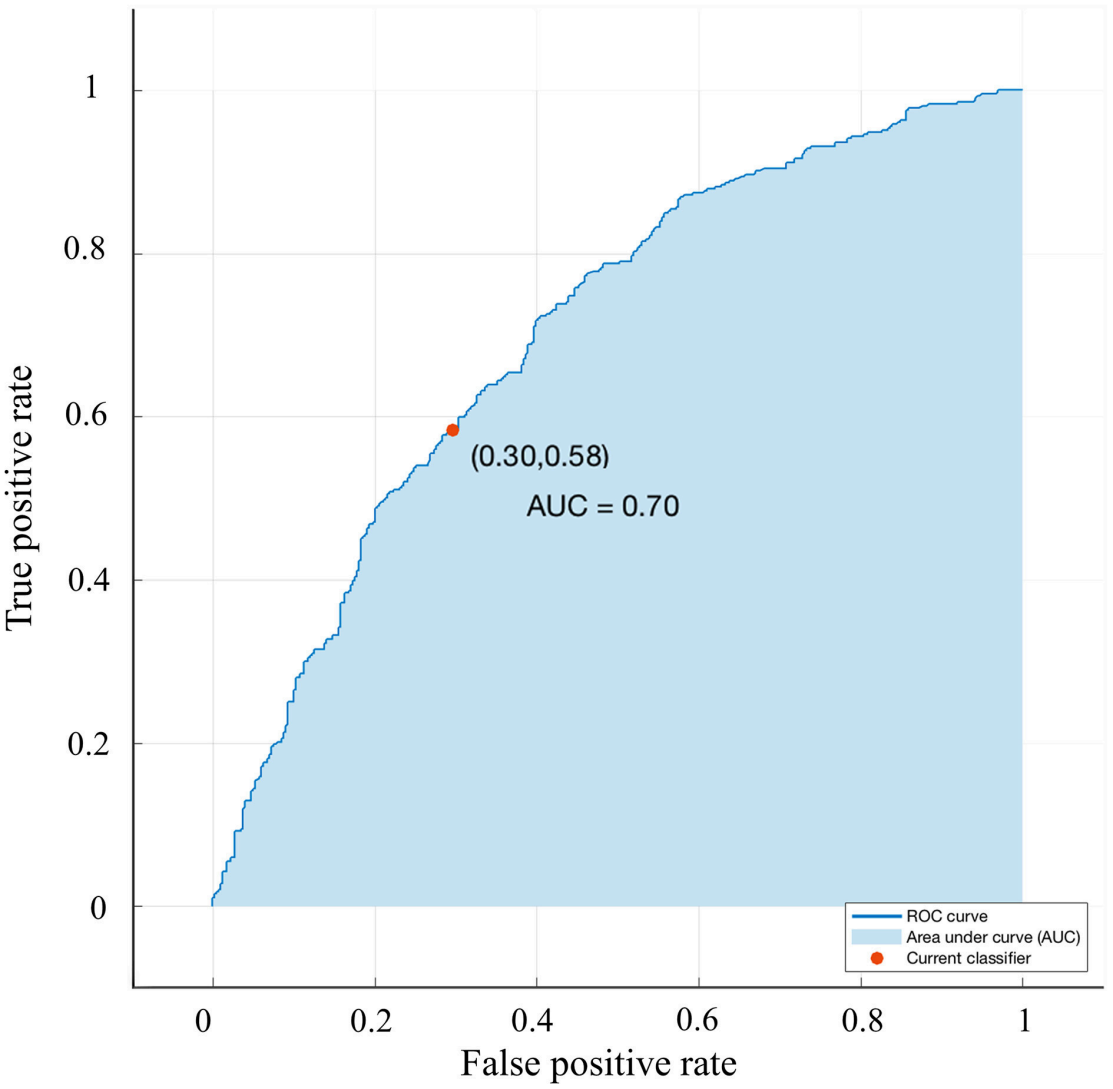
Validation using a ROC curve. Established by Smote-Bagged-tree and used Simply using quadrant method to analyze the location of the tumor. The AUC value is 0.701.

**Figure 8:**
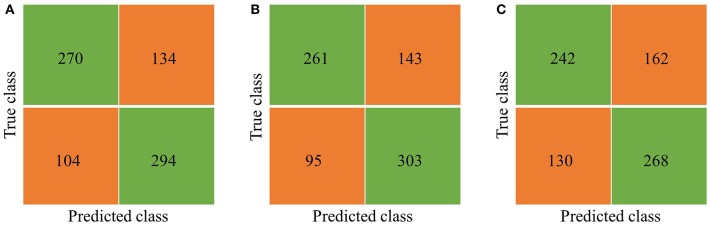
Confusion matrix. **(A)** Vector combining quadrant method, the Accuracy is 70.3%. **(B)** Simply using vector method, the Accuracy is 70.3%. **(C)** Simply using quadrant method, the Accuracy is 63.6%.

A bagged tree was used to categorize samples. In order to obtain a reliable and stable model, a 5-fold cross-validation was used for verification. A Sentinel lymph node prediction program was established by SMOTE-bagged tree algorithm. Since the original location of the tumor was analyzed by the combination of vector and quadrant for the first time, we used the method of simply using quadrant or vector for comparison. Finally, our model obtained the AUC value of 0.801 (Figure 5), while the vector group is 0.791 (Figure 6) and the quadrant group is 0.701 (Figure 7). We use the confusion matrix to evaluate the accuracy of the model. The accuracy of our model is 70.3% (Figure 8A), compared to 70.3% for the vector group (Figure 8B) and 63.6% for the quadrant group (Figure 8C).The mentioned method provides an accurate and credible multi-variable prediction model.

The inclusion of imaging and pathological detection factors in an easy-to-use machine learning model facilitates the prediction of lymph node metastasis in patients before surgery. Because lymph node metastasis is similar in training set and validation set, a predictive model for breast cancer lymph node metastasis based on Bagged Tree algorithm is of great importance, and can be directly applied to verify dataset. The experimental results showed that the prediction model for lymph node metastasis on the basis of SMOTE-Bagged Tee algorithm can effectively improve the rate of data utilization and assist doctors predict sentinel lymph node metastasis in breast cancer patients.

In order to make it easier to use in clinical applications, we have created an app that patients can use it easily by a personal computer, laptop, or smart phone. The operation method is simple, only the user should input the patient's clinicopathological information into the app.

## Discussion

The prediction results obtained with the help of a predictive model are more credible than simple clinical guesses. The development of each predictive model is accomplished through the clinicopathological data of different populations. In our model, in terms of tumor size, we chose T1-T2 staging patients, because for the study of exemption from SLNB, the need for early clinical staging of the patient group, thus the size of tumor needs to be strictly limited. For type of tumor, we focused on invasive ductal carcinoma, which accounts for only a small fraction of lobular cancer and mucous cancer. As invasive ductal carcinoma accounts for the vast majority, in order to avoid the formation of bias, the type of tumor was not taken into account in the inclusion of variables. Because chemotherapy can affect lymphatic vessels, SLNB has a high failure rate and a high false negative rate, hence, we ruled out patients with preoperative neoadjuvant chemotherapy. There were two variables for nerve infiltration and vascular thrombosis, and because the selected cases were early breast cancer patients, the number of cases in these two states was very limited. Moreover, these two variables could only be learned by breast cancer surgery, and the establishment of a preoperative prediction model was not practical, thus the variables of the model were not included.

In several studies, tumor size is the main predictor of sentinel lymph node metastasis (9–14). According to the results of statistical analysis, we also confirmed that the rate of positive SLN was lower in the group of patients with smaller tumors. This is also consistent with the results of NSABP-04, ASCOG-Z0011, IBCSG 23-01, and other tests. Axillary lymph node dissection (ALND) is not necessary for early breast cancer patients with 1 to 2 positive SLNs after undergoing lumpectomy, radiotherapy (RT), and systemic treatment (3, 4, 27–29).

We compare and analyze the predictive ability of smote-bagged tree algorithm modeling and Logistic regression method modeling, and the results suggest that the model established by smote-bagged tree algorithm is more predictive. The main reasons are the following: (a) Because of the imbalance of data, using the Bagged-Tree algorithm can reduce the impact of data imbalance. It performs well on the category imbalance data and improves the prediction accuracy. (b) Feature normalization or standardization is not required. Especially when the scale of the feature is completely different or when the binary feature and the continuous feature exist simultaneously, the effect is very good.

To our knowledge, this is the first model analyzing and studying the original location of the tumor by both vector and quadrant. We analyzed the previously established axillary lymph node prediction models (8–15, 17–20), and found that there were different views on the effects of the primary location of the tumor on sentinel lymph node metastasis. The MSKCC model concluded that the risk of axillary lymph node metastasis in the upper inner quadrant was less, while there was no statistically different chance of axillary lymph node metastasis between other quadrants (11). In 2012, the SCH model proposed by Chinese scholars mentioned that in terms of the location of the tumor, the order of axillary lymph node metastasis from high to low should be the central quadrant, the lower inner quadrant, the outer upper quadrant, the lower outer quadrant, and the upper inner quadrant (20). This study takes into account the breast as a three-dimensional (3D) structure, thus we first proposed the primary location of the tumor through both vector and quadrant location analysis. By comparing the three methods, the results prove that: We used the vector combining quadrant method to analyze the original location of the tumor, which is more precise than simply using vector or quadrant. The combination of the two methods, the primary location of the tumor will be more precisely positioned.

BMI was used as an important factor in previous researches (11, 20, 30). To our knowledge, breast composition includes fat and glands, and BMI does not indicate the size of breast glands in the overall proportion of the breast, thus our study for the first time presents the breast gland content as an independent factor for analysis.

Breast malignant tumor has its special form; for example, ultrasound is typically classified through the morphology, boundaries, activity, and other conditions of the tumor; molybdenum target will be classified according to the shape of the tumor and calcification (31). In retrospect, the morphology of the tumor was not analyzed as a factor in previous studies. Because the judgment of ultrasound is subjective, we use the tumor morphology under molybdenum target as a factor for statistical analysis. After deep learning of the image by computer, the picture of the breast molybdenum target image is automatically identified, and the shape of the tumor is scientifically grouped, and that is used as an important factor in the production of predictive software.

Looking back at the predictive models of other researches, the variables included in each model are different, and we believe that a predictive model should be simplified on the basis of ensuring accuracy, rather than only simply pursuing variables. Our predictive model contains 12 variables: age, BMI, quadrant, clock direction, the distance of tumor from the nipple, morphology of tumor molybdenum target, glandular content, tumor size, ER, PR, HER2, and Ki-67. Generally speaking, when the AUC value of a model is at the range of 0.7–0.8, the prediction ability of the model is superior. When the AUC value is at the range of 0.8–0.9, the prediction ability of the model is very good. Through the SMOTE-Bagged Tree algorithm, the AUC value obtained by our model is 0.80, which proves that our model has a proper prediction ability, and can be used for early breast cancer patients.

Our SLN prediction model appropriately predicts the risk of sentinel metastasis in patients. For patients with low risk of SLN metastasis, especially those who cannot tolerate SLNB surgery, or patients with high retention requirements for postoperative limb sensory function, no SLNB can be considered clinically to improve the patients' quality of life. For patients with high risk of SLN metastasis, axillary treatment can be directly performed, such as ALND, axillary radiotherapy, etc., especially for elderly or patients with poor foundation status, that can greatly shorten the duration of operation time. For patients undergoing neoadjuvant chemotherapy, this model can be used to predict its SLN state before the new auxiliary chemotherapy, and our model does not include the patients after the new adjuvant radiotherapy, and does not apply to the prediction after the new adjuvant chemotherapy, limiting the prediction ability of the model.

In clinical work, more and more patients are eager to understand the pathological state of SLN before surgery. Predictive results obtained using objective predictive models are more believable than pure clinical guesses. According to the sentinel lymph node prediction model established by this research data, the overall prediction ability is very high, the result of the ROC Curve area is 0.801, which suggests that our prediction model has good predictive ability and strong stability, so we believe that the model can be generally applied to other groups of people. Compared with other models, we used the factors for the first time as follows: the location of the tumor was analyzed by vector combining quadrant method, the content of the breast glands, and the shape of the tumor, which caused that our model to be more sophisticated. Since the variables required by the model can be obtained by ultrasound, molybdenum target, hollow core needle biopsy, or open biopsy, the patient is able to learn the risk of SLN metastasis before the operation to predict the prognosis of the disease.

However, our predictive model contains some limitations. Firstly, in the next study, for the variant of tumor morphology under molybdenum target, we should incorporate more data, and continue to improve the depth of machine learning, so that it can be used to more detailed grouping of tumor morphology, and strive to further identify calcification patterns. Secondly, the breast is such a 3D structure, in which for finding the location of the tumor, we will then improve its grouping, and strive to complete the 3D positioning. In addition, this is a retrospective and single-center study, and out model has a proper diagnostic ability, however, it needs to be further validated in other regions and populations.

## Conclusions

In summary, we have established an accurate, reliable, and user-friendly multi-variable predictive model. By adding several variables that have never been used in previous models, our model can be used to predict the risk of sentinel lymph node metastasis before breast cancer surgery, and it provide a reliable basis for the treatment of axillary lymph nodes.

## Ethics Statement

The study was granted ethical approval by the Ethical Committee of China Medical University and the Shengjing Hospital of China Medical University. All the patients provided written informed consent.

## Author Contributions

ChL, ZZ, XG, YZ, XC, and CaL contributed conception and design of the study. XG, LS, GC, HZ, and YJ organized the database. ChL, ZZ, and LS performed the statistical analysis. ChL and ZZ wrote the first draft of the manuscript. ChL, ZZ, XG, YZ, XC, and CaL wrote sections of the manuscript. All authors contributed to manuscript revision, read and approved the submitted version.

### Conflict of Interest Statement

The authors declare that the research was conducted in the absence of any commercial or financial relationships that could be construed as a potential conflict of interest.

## Abbreviations

SLN: Sentinel lymph node
SLNB: Sentinel lymph node biopsy
SLN+: Sentinel lymph node positive
SLN-: Sentinel lymph node negative
ALN: Axillary lymph node
ALND: Axillary lymph node dissection
MLR: Multiple logistic regression
SMOTE: Synthetic minority oversampling
UIQ: Upper inner quadrant
UOQ: Upper outer quadrant
LOQ: Lower outer quadrant
LIQ: Lower inner quadrant
Central: Central quadrant
ROC: Receiver operating characteristics curve
AUC: Area under the receiver operating characteristics curve
BMI: Body mass index
IHC: Immunohistochemistry
ER: Estrogen receptor
PR: Progesterone receptor
HER2: Human epidermal growth factor receptor 2.
